# Ligand-Induced U Mobilization from Chemogenic Uraninite and Biogenic Noncrystalline U(IV) under Anoxic Conditions

**DOI:** 10.1021/acs.est.1c07919

**Published:** 2022-05-06

**Authors:** Kyle J. Chardi, Anshuman Satpathy, Walter D. C. Schenkeveld, Naresh Kumar, Vincent Noël, Stephan M. Kraemer, Daniel E. Giammar

**Affiliations:** † Centre for Microbiology and Environmental Systems Science, Department for Environmental Geosciences, University of Vienna, Josef-Holaubek-Platz 2, 1090 Vienna, Austria; ‡ Department of Civil and Environmental Engineering and Earth Sciences, University of Notre Dame, Note Dame, Indiana 46556, United States; § Soil Chemistry and Chemical Soil Quality Group, Wageningen University and Research, Droevendaalsesteeg 3, 6708 PB Wageningen, The Netherlands; ∥ Stanford Synchrotron Radiation Lightsource, SLAC National Accelerator Laboratory, 2575 Sand Hill Road, Menlo Park, California 94025, United States; ⊥ Department of Energy, Environmental, and Chemical Engineering, Washington University, One Brookings Drive, St. Louis, Missouri 63130, United States

**Keywords:** uranium, chelating ligands, mobilization, monomeric U(IV), ion-exchange chromatography, dissolution kinetics, U redox speciation

## Abstract

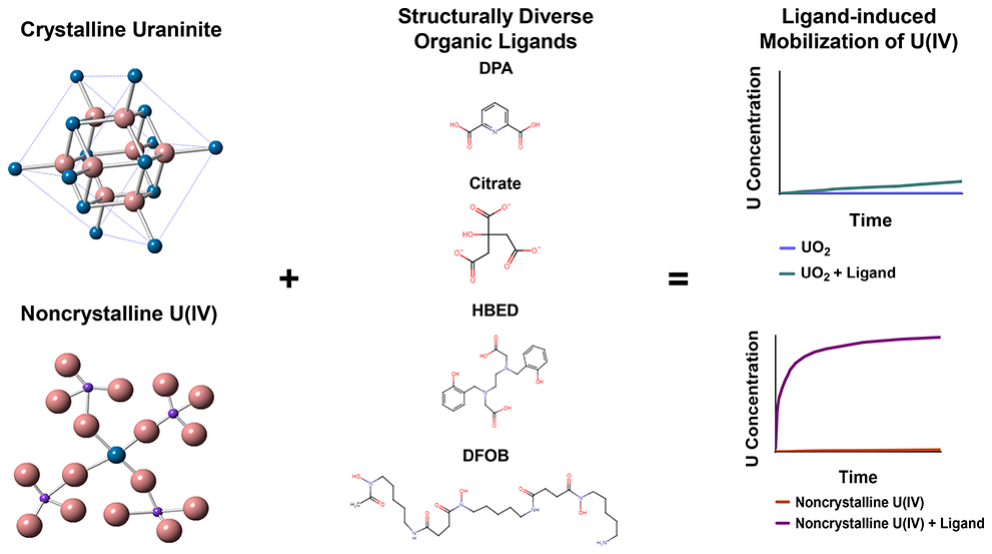

Microbial reduction of soluble hexavalent uranium (U(VI)) to sparingly soluble tetravalent uranium (U(IV)) has been explored as an *in situ* strategy to immobilize U. Organic ligands might pose a potential hindrance to the success of such remediation efforts. In the current study, a set of structurally diverse organic ligands were shown to enhance the dissolution of crystalline uraninite (UO_2_) for a wide range of ligand concentrations under anoxic conditions at pH 7.0. Comparisons were made to ligand-induced U mobilization from noncrystalline U(IV). For both U phases, aqueous U concentrations remained low in the absence of organic ligands (<25 nM for UO_2_; 300 nM for noncrystalline U(IV)). The tested organic ligands (2,6-pyridinedicarboxylic acid (DPA), desferrioxamine B (DFOB), *N*,*N*′-di(2-hydroxybenzyl)ethylene-diamine-*N*,*N*′-diacetic acid (HBED), and citrate) enhanced U mobilization to varying extents. Over 45 days, the ligands mobilized only up to 0.3% of the 370 μM UO_2_, while a much larger extent of the 300 μM of biomass-bound noncrystalline U(IV) was mobilized (up to 57%) within only 2 days (>500 times more U mobilization). This work shows the potential of numerous organic ligands present in the environment to mobilize both recalcitrant and labile U forms under anoxic conditions to hazardous levels and, in doing so, undermine the stability of immobilized U(IV) sources.

## Abstract 2

Ligand-induced mobilization of U from uraninite and, to a greater extent, noncrystalline U(IV) was shown to appreciably increase dissolved U concentrations.

## Introduction

Uranium (U) is the most abundantly found radionuclide in groundwater, soils, and sediments at U.S. Department of Energy (DOE) contaminated sites^1^ and poses environmental and human health threats globally. Hazardous levels of U in the environment result from both anthropogenic activities and geogenic processes, which can lead to U concentrations exceeding World Health Organization (WHO) guidelines for drinking water limits (30 μg L^−1^).^2−4^ Uranium mobility is strongly affected by its oxidation state, with hexavalent uranium (U(VI)) species being significantly more soluble than tetravalent uranium (U(IV)) species.^5^ Microbial reduction of soluble U(VI) to sparingly soluble U(IV) has been tested as an *in situ* bioremediation strategy for the immobilization of U in contaminated aquifers,^6−8^ such as the Rifle, Colorado site.^9−13^

Oxidative mobilization is a primary source of concern to maintaining immobilization of U(IV) in remediation efforts. Uraninite (UO_2_) is one of the poorly soluble U(IV) phases that can form and has been found in remediated sediments. It is also a major constituent of ores and spent nuclear fuel placed in geologic repositories.^14−17^ In nature, UO_2_ is typically oxidized to some extent and thus can be denoted as UO_2+*x*_ to account for the additional oxygen.^18,19^ Dissolved oxygen and nitrate facilitate UO_2_ dissolution through oxidative processes on the mineral surface.^20−22^ Previous studies have reported that even under reducing conditions, UO_2_ surface sites can react with water (as an oxidant) to form U(V) and U(VI).^16,21^

Recent research has shown that the product of U(VI) bioreduction additionally includes noncrystalline U(IV) species.^23,24^ Noncrystalline U(IV) is operationally defined as U(IV) solid species that lack the U–U pair correlation characteristic of U(IV) minerals in extended X-ray absorption fine structure (EXAFS) Fourier transform spectra and that lack a crystalline lattice.^23^ In many cases, noncrystalline U(IV) is bound to phosphate or carboxyl groups of biomass.^23,25^ Noncrystalline U(IV) has been identified in numerous field locations including naturally U-rich peat soils,^26^ wetlands impacted by U mining activities,^24^ naturally reduced sediments,^15^ and remediated sediments.^27−29^ Furthermore, bioreduced sediments containing noncrystalline U(IV) were shown to not alter their U(IV) speciation or crystallinity in aging studies (over 12 months), illustrating its persistence in the environment and long-term vulnerability to remobilization.^30,31^

Noncrystalline U(IV) has been found to be substantially more labile than UO_2_ due to its propensity to be readily mobilized by bicarbonate and oxidized by O_2_ and persulfate.^25,32,33^ In previous studies with noncrystalline U(IV), some U(VI) was found to be associated with the noncrystalline U(IV) even after bicarbonate extraction.^33^ These recent findings regarding the susceptibility of U(IV) solubilization raise questions pertaining to the mobility of reduced U species in the environment. A few studies have drawn direct comparisons between U(IV) phases with differing labilities,^32,34^ and there are currently no studies that directly compare the effects of organic ligands on UO_2_ and noncrystalline U(IV). Furthermore, only a few studies have investigated the role of chelating ligands in mobilizing U from reduced U(IV)-containing solid phases. Desferrioxamine B (DFOB) has been shown to increase UO_2_ dissolution rates under anoxic conditions.^35^ Additionally, citrate and ethylenediaminetetraacetic acid (EDTA) were shown to increase the solubility of both U(IV) and U(VI) in anoxic field sediments.^36^

The findings of differing labilities across U species under anaerobic conditions and the potential for organic ligands to enhance the mobilization of various metals in the environment highlight a knowledge gap pertaining to the mobility and reactivity of (partly) reduced U phases in the environment, specifically in regard to mobilization by organic ligands. The aims of this study were (1) to systematically evaluate and quantify the effectiveness of different organic ligands in mobilizing U from UO_2_ and (2) to gauge the propensity for ligand-induced U mobilization from noncrystalline U(IV) and elucidate the differences compared to UO_2_. Anoxic batch experiments were carried out at varying ligand concentrations with U(IV)-bearing phases to assess their reactivities in the presence of organic ligands. The results obtained here provide a deeper understanding of the mobility and reactivity of U in the environment under anoxic conditions, highlighting the implications of U complexation by organic ligands, which can contribute to the scientific basis for the design of remediation strategies and predictions of U mobility in contaminated subsurface environments and geologic repositories.

## Materials and Methods

### Material Synthesis

Chemogenic UO_2_ was synthesized following the protocol described in Ulrich et al., for which the details are described in full in the Supporting Information (SI).^34^ Briefly, studtite (UO_2_O_2_·4H_2_O_(s)_) was precipitated by mixing H_2_O_2_ with UO_2_Cl_2_ and subsequently reduced to UO_2_ by H_2(g)_ at 400 °C for 4 h. Noncrystalline U(IV) was synthesized as described in Bernier-Latmani et al.^23^*Shewanella oneidensis* MR-1 cultures were grown in sterile Luria-Bertani broth (LB medium) until midexponential phase. Cells were then harvested by centrifuging at 7000 RCF (relative centrifugal force) for 10 min and washed in a sterile anoxic phosphate-containing medium (Widdel Low Phosphate, WLP) before being resuspended in WLP amended with piperazine-*N*,*N*′-bis(2-ethanesulfonic acid) (PIPES) buffer adjusted to pH 7.0, bicarbonate, lactate, and 0.5 mM U standard in 5% HCl (uranyl nitrate evaporated and resuspended in HCl) (Table S1).^37^ After 14 days of reduction in air-tight glass serum bottles, dissolved U concentrations in the suspensions were measured to confirm the extent of U removal from solution (>99.99% total U removed). Immobilization was therefore the result of U association with biomass (see below).^38^

Deionized water (DI water, resistivity >18.2 MΩ·cm, Milli-Q, Millipore) was used for all solutions and suspensions. Anoxic DI water (O_2_ conc. < 1 ppmv) was purged with N_2(g)_ for 6 h before being brought into an anaerobic chamber where it equilibrated overnight before use.

### Chemogenic UO_2_ and Biogenic Noncrystalline U(IV) Characterization

X-ray powder diffraction (XRD, Bruker d8) was used to characterize the freshly prepared UO_2_; method detailed in the SI. The XRD pattern for UO_2_ aligned well with the International Center for Diffraction Data ICDD reference pattern (ICDD 00–041–1422) (Figure S1).

The specific surface area (SSA) of UO_2_ was estimated based on its density, and the size and shape of the UO_2_ crystals were determined using scanning electron microscopy (SEM, FEI Nova Nano 230) (Figure S2). Fifty particles were measured using the imaging software ImageJ to determine an average particle size of 112 nm (standard deviation = 13 nm). Particles were assumed to have a cubic shape (based on SEM imaging) and a density of 10.99 g cm^–3^, consistent with published data.^34,39^ The calculated SSA of 4.9 m^2^ g^–1^ is also consistent with the literature from Brunauer–Emmett–Teller (BET) and SEM analyses.^34,40^

X-ray absorption spectroscopy was used to characterize noncrystalline U(IV) after bicarbonate rinsing (detailed below) (Figures S3 and S4). Details are provided in Text S3.

### Ligand Mobilization Experiments

The effect of organic ligands on U mobilization was studied for chemogenic UO_2_ and biogenic noncrystalline U(IV) under nonsterile conditions at U concentrations of 370 μM (0.1 g L^–1^ UO_2_) and 300 μM, respectively. For UO_2_ experiments, ligand concentrations of 5 μM, 50 μM, 500 μM, and 2 mM were aimed to bracket the estimated available surface site concentrations. The lowest ligand concentration (5 μM) was comparable to the surface site concentration of 1.3 μM sites (based on an SSA of 4.9 m^2^ g^–1^, suspension density (SSR) of 0.1 g L^–1^ UO_2_, and site density of 2.3 sites nm^–2^ applied as an estimate for metal oxides).^41^ The 500 μM ligand concentration was comparable to the total U concentration in the system (1:1 U:ligand concentration of 370 μM) while the 2 mM ligand concentration was in excess of the total U. For noncrystalline U(IV) experiments, ligand concentrations of 5 μM and 2 mM were used; the lowest and highest concentration in the UO_2_ experiments. Ligand-free control treatments for both chemogenic UO_2_ and biogenic noncrystalline U(IV) experiments were conducted in duplicate over two distinct separate experiments and across four reactors (*n* = 4). Time *t* = 0 corresponded to the time of addition of the well-mixed U(IV) phase stock suspension to the buffer/electrolyte/ligand solution. Digestions of stock suspensions were found to be within 1 μM U of intended stock concentration. Suspensions were continuously mixed on an orbital shaker.

The current study focused on four organic ligands that were selected because of differences in their number and type of functional groups. 2,6-pyridinedicarboxylic acid (DPA), a compound with pyridine and carboxylate functional groups, is a low-molecular-weight organic acid that is a natural product of bacterial sporulation. It provides protection from unfavorable environmental conditions^42^ and constitutes up to 15% of the dry weight of bacterial spores.^43^ Desferrioxamine B (DFOB), a trishydroxamate, is a microbial siderophore.^44^ Synthetic chelators like *N*,*N*′-di(2-hydroxybenzyl)ethylene-diamine-*N*,*N*′-diacetic acid (HBED) are often released into the environment in industrial or agricultural contexts. HBED contains phenolate, amine, and carboxylate groups. Bioavailable Fe-HBED complexes are commonly used in agriculture in Fe fertilizers to mitigate plant iron deficiency due to poor solubility of Fe-(hydr)oxide minerals at circumneutral pH, particularly in calcareous soils.^45^ Citrate, a triscarboxylate, is a low-molecular-weight organic acid that is ubiquitous in nature and present in some radioactive waste sites.^46,47^ The hexadentate ligands with hard phenolate or hydroxamate Lewis base groups (HBED and DFOB) were expected to have a high affinity for U(IV) (as a hard acid), whereas ligands of lower denticity lacking the aforementioned hard Lewis base groups were expected to be less effective at chelating and mobilizing U(IV).

Mobilization experiments were conducted in an anaerobic (O_2_ conc. < 1 ppmv) chamber (Coy Laboratory Products, Inc.) containing a gas mixture of 95% N_2(g)_ and 5% H_2(g)._ Batch reactors in duplicates (polypropylene, 100 mL for UO_2_; glass, 30 mL for noncrystalline U(IV)) were aluminum foil wrapped to prevent photochemical reactions. The pH of all solutions and suspensions was continuously monitored (Accumet XL 15, Fisher).

The pH was buffered to pH 7.0 (within the typical groundwater pH range of 6–8.5) using 10 mM 3-(*N*-morpholino) propane sulfonic acid (MOPS, p*K*_a_ = 7.28). MOPS was used previously in UO_2_ dissolution studies with no observed effect on U mobilization.^32,35,40,48^ Adjustments to pH were done by adding aliquots of NaOH or HCl solution prior to the addition of UO_2_ or noncrystalline U(IV). NaCl was added as an electrolyte to a final ionic strength of 0.01 M (carbonate-free).

Stock suspensions of each U phase were prepared at 100 times concentrated in an anaerobic chamber for each set of experiments (37 mM U for UO_2_; 30 mM U for noncrystalline U(IV)). Prior to the start of each experiment, the U material was washed in a solution of anoxic NaHCO_3_ (1 M for 10 h for UO_2_, 50 mM for 10 h for noncrystalline U(IV)) to extract any initially present U(VI) from the material surfaces.^38^ Due to the larger lability of noncrystalline U(IV), a lower HCO_3_^–^ concentration was used as in previous studies.^32,33,38^

The suspension was then centrifuged at 7000 RCF for 10 min in Nalgene Oak Ridge centrifuge tubes (PP) with sealing caps (similar to other studies in the literature).^25,32,33,38^ The supernatant was decanted, and the solids were resuspended in anoxic DI water. This procedure was repeated four times to remove residual HCO_3_^–^ from the cleaning step. A subsample (in duplicate) was taken from the suspension and digested in 10% HNO_3_ at 100 °C for 4 h and analyzed by inductively coupled plasma mass spectrometry (ICP-MS) to determine the exact stock suspension concentration. Once each batch reactor was prepared (ligand added and ionic strength and pH fixed), aliquots of the (continuously stirred) stock U suspension were spiked into the reactors.

Reactors were sampled over time and UO_2_ samples were filtered with 0.025 μm mixed cellulose ester (MCE) membrane filters (25 mm diameter PP filter holder), while for noncrystalline U(IV), 0.2 μm cellulose acetate filters were used (only minor differences in dissolved U concentrations noted in filter tests). Samples were acidified with trace-metal-grade HNO_3_ for analysis of dissolved U concentrations by ICP-MS.

An ion-exchange chromatography method for resolving U oxidation state^29,49^ (method detailed Text S4) was used on the end-point samples for both ligand concentrations with noncrystalline U(IV) (not carried out for UO_2_ due to limit of quantification limitations). Several studies including noncrystalline U(IV) with organic ligands have previously used the method.^33,50^

### Mobilization Rate Calculation

Uranium mobilization rates for chemogenic UO_2_ were calculated (as sufficient sample points over time were available to establish rates and due to the lack of a discrete surface to quantify the SSA of noncrystalline U(IV)) for all ligand treatments to compare the kinetics of U release using a linear regression approach. Net mobilization rates were determined without considering U readsorption as U complexes, thereby, potentially underestimating the release of U from the UO_2_ structure.^51^ Rates were calculated for all ligands in two stages: from 0 to 2 days and from 2 to 23 days of the experiment (total experiment length = 45 days) to compare the initial faster mobilization rates to the slower steady release later in the experiment (rates and corresponding coefficient of determination, *R*^2^, summarized in Table S3). Comparisons to rates of noncrystalline U(IV) mobilization were carried out by determining the total extents of U mobilization over the experimental duration for UO_2_ and noncrystalline U(IV), respectively.

### Equilibrium Speciation Calculations

Equilibrium UO_2_ solubility (UO_2(am)_) under experimental conditions was calculated in Visual MINTEQ. Except citrate, ligands used in this study do not have reported stability constants with U(IV). In the case of citrate, models were prepared based on known constants^52−54^ (Figure S5). In light of the ease with which UO_2_ can undergo minor surface oxidation to U(V) and U(VI) even in the absence of O_2_ by reaction with water,^19,21^ models were additionally prepared with U(VI) to investigate its possible role in ligand mobilization. With exception of HBED, complexation constants for U(VI) are available.^53,55,56^ Aqueous speciation models were prepared to predict U speciation in the presence of the ligands used in this study under the assumption that all of the mobilized U was U(VI) (Figure S6). This allowed for assessing the feasibility of U(VI) speciation accounting for an appreciable extent of U mobilization in batch experiments. All constants used in the model are summarized in Table S2.

## Results and Discussion

### Ligand-Induced Mobilization of Chemogenic UO_2_

**Figure 1:**
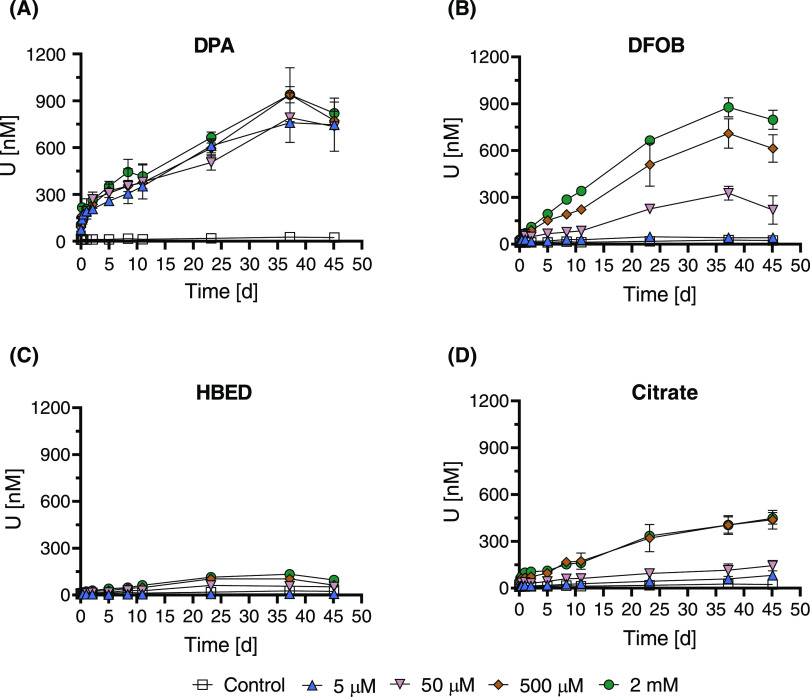
U mobilization from chemogenic UO_2_ (370 μM U) induced by (A) DPA, (B) DFOB, (C) HBED, and (D) citrate at concentrations of 5 μM, 50 μM, 500 μM, and 2 mM with a fixed pH of 7.0. All experiments included 10 mM MOPS buffer and 6.6 mM NaCl. Control treatments had the same composition as other treatments with the exception of the ligand. Error bars indicate standard deviations of duplicate reactors. Time *t* = 0 h corresponds to the moment of addition of an aliquot of the UO_2_ stock suspension to the solution containing the pH buffer, electrolyte, and ligand.

Batch experiments were conducted to probe the rate and extent of ligand-induced U mobilization from chemogenic UO_2_ by various organic ligands (Figure 1A–D). Over the 45-day experiment, dissolved U concentrations in ligand-free control treatments remained relatively constant, ranging between 10 and 25 nM. These values are within an order of magnitude of the predicted equilibrium U concentration of 3 nM for the solubility of UO_2(am)_ under experimental conditions. Small deviations from predicted U concentrations may be attributed to minor differences in *K*_sp_ between the synthesized material and that reported in the literature (Table S2). Also, possible surface oxidation of U(IV) to U(V) or U(VI) surface species by water (after 1 M NaHCO_3_ rinsing prior to starting the experiment) as reported previously, and subsequent partitioning of U(VI) into solution may have resulted in a somewhat larger soluble U concentration than predicted from UO_2_ solubility.^21^

**Figure 2:**
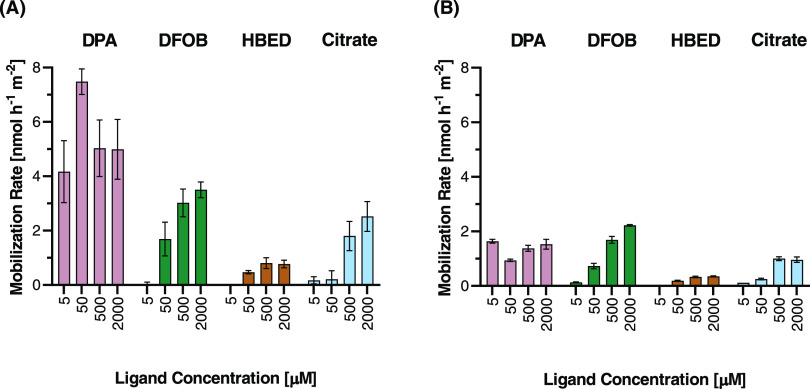
U mobilization rate from chemogenic UO_2_ for all applied ligand concentrations for DPA, DFOB, HBED, and citrate. Error bars represent standard error of linear regression analysis from batch mobilization data. All rates are calculated over two segments: (A) the initial fast mobilization rate from 0 to 2 days and (B) the later linear stage before beginning to level off from 2 to 23 days. The U mobilization rate in control treatments was 0.03 nmol h^–1^ m^–2^ in both stages.

The four tested ligands mobilized U to varying extents and at differing rates. Ligand-induced U mobilization from UO_2_ began with a faster initial mobilization followed by a slow-release mechanism, remaining relatively linear over the experimental duration until the final sample points, where dissolved U concentrations for all ligands except citrate appeared to begin leveling off. Notably, experiments with DPA exhibited a substantially more distinct initial swift mobilization than experiments with the other ligands. Calculated mobilization rates were normalized to UO_2_ surface area as well as mass (Table S3) and plotted for all applied ligand concentrations (Figure 2).

#### DPA

DPA (Figure 1A) was the most effective ligand at mobilizing U from UO_2_, especially at low ligand concentrations. It mobilized over 750 nM U with only 5 μM DPA addition. DPA exhibited a unique result of showing minimal ligand concentration dependency, mobilizing <200 nM additional U at a ligand concentration of 2 mM compared to at 5 μM. While ligand concentration independence of dissolution rates could be explained by saturation of UO_2_ surface sites already being achieved by the addition of 5 μM DPA, this would not explain the independence of maximum dissolved uranium concentrations observed toward the end of the dissolution experiment, assuming close to equilibrium conditions were reached. The comparatively large U mobilization by DPA was surprising, as DPA was not expected to be particularly effective at mobilizing U(IV) (redox state of the mobilized U was not analyzed). While the existence of 1:2 and 1:3 U(IV) DPA complexes has been established in the literature, stability constants for U(IV)-DPA complexes that would be needed to quantitatively predict the effect of DPA on UO_2_ solubility are not available.^57−59^

The formation of U(VI)-DPA complexes has been studied more intensively, and stability constants for 1:1 and 1:2 complexes are available.^56,60,61^ Previous studies on U(VI)-DPA and Np(V)-DPA complexation both attributed the strong complexation between each respective actinide and DPA to the rigid structure of DPA in minimizing the preorganization energy required for complexation.^56,62^ Equilibrium models describing the aqueous U speciation in the presence of DPA under the assumption that all mobilized U (Figure 1A) was in fact U(VI) predicted >99.5% of the U(VI) to be complexed by DPA at each DPA concentration (Figure S6). This illustrates the ability of DPA complexation to dominate U(VI) speciation under experimental conditions.

Hence, DPA may be an effective scavenger of U(VI) that was generated through surface oxidation of U(IV) by water, as documented in previous studies.^21,63^ This would be consistent with the observed independence of the U mobilization rate on the DPA concentration, suggesting that U mobilization rates are controlled by the oxidation kinetics of U(IV) at the mineral surface. The rapid initial U mobilization could then correspond to the potential U(VI) generated between the bicarbonate wash and the addition of the DPA. Once DPA was added, residual U(VI) would have been scavenged into solution (fast initial release) (Figure 2A). The following gradual increase in dissolved U concentration would then be driven by the ongoing surface oxidation of U(IV) and subsequent complexation by DPA (Figure 2B). This interpretation points toward a mechanism where trace quantities of surface-associated U(VI) could be rapidly mobilized from the UO_2_ surface by a ligand with a high affinity for U(VI) (such as DPA) even under strictly anoxic conditions, which has previously been confirmed in the literature that U(V) and U(VI) can reside on the UO_2_ surface through radiolysis and hydrolysis reactions.^19,21^

#### DFOB

The rate and extent of DFOB-induced U mobilization from UO_2_ increased with increasing ligand concentration (Figure 1B). A clear ligand concentration dependency was seen on both the total extent of U mobilized as well as the mobilization rate, with both increasing with each successive increase in ligand concentration from 5 μM up to 2 mM DFOB. An elevated initial dissolution rate over the first two days was observed, but the difference in the second phase dissolution was much smaller than that observed for DPA.

Frazier et al. found a linear relationship between steady-state UO_2_ dissolution rates and adsorbed DFOB concentrations suggesting a ligand-promoted dissolution mechanism.^35^ They reported that DFOB adsorption onto UO_2_ follows a Langmuir-type relationship with a maximum adsorbed DFOB concentration of 3.3 μmol m^–2^.^35^ The Langmuir isotherm from Frazier et al. was used for relating the DFOB surface concentrations to the observed mobilization rates, and subsequently using their rate law while accounting for the experimental parameters of our setup (SSA and SSR). We also observe a roughly linear relationship (*R*^2^ = 0.994 and 0.978 for 0–2 days and 2–23 days stages, respectively) between adsorbed ligand concentrations and mobilization rates (Figure S7), again suggesting a ligand-promoted dissolution mechanism in which the ligand surface concentration is rate controlling.^64^ It is important to note, however, that Frazier et al. found a higher dissolution rate constant under steady-state conditions (1.90 × 10^–2^ h^–1^) compared to our estimated rate constant *k*_L_ of 1.04 × 10^–3^ and 6.02 × 10^–4^ h^–1^ observed during the initial (0–2 days) and later stages (2–23 days) of dissolution, respectively. The substantially higher rate constant found by Frazier et al. is hypothesized to be due to the different mineral synthesis procedure, producing a less crystalline product as indicated by less defined and broader peaks in the XRD pattern than for the material used in the present study. The less crystalline product would in turn have increased solubility and dissolution rates.

Electrostatic attraction occurs between the UO_2_ surface (point-of-zero-charge (PZC) = 5.4) and DFOB at pH 7.0 (p*K*_a1_ of DFOB = 8.3; dominant species: H_4_DFOB^+^). For the other ligands in the study, electrostatics are less favorable for adsorption as they are negatively charged at pH 7.0, similar to the UO_2_ mineral surface. Yet Frazier et al. found DFOB adsorption to UO_2_ to be constant between pH 3 and pH 8 despite the change in the surface charge of UO_2_, suggesting that electrostatic interactions only impart a small contribution to the energetics of DFOB adsorption to the UO_2_ surface.

#### HBED

The phenolate-containing chelator HBED (Figure 1C) only minimally enhanced UO_2_ dissolution with dissolved U concentrations peaking at <150 nM U for 2 mM HBED. This was surprising since the hard phenolate ligating groups are expected to have a high affinity for U(IV).^65^ Also, considering that U(IV) has a greater charge-to-radius ratio and first hydrolysis constant than Fe(III) and noting that HBED is a strong complexing agent for Fe(III), a high affinity to U(IV) would be expected.

It is notable that, while U mobilization was dependent on the HBED concentration, at the lowest ligand concentration (5 μM), HBED inhibited U release to solution (Figures S8 and 2). If the total dissolved U concentration in ligand-free treatments were governed by UO_2_ solubility, the addition of ligands would not decrease it. Therefore, the addition of HBED may have shifted the partitioning of U(VI) originating from surface oxidation toward more surface-associated U(VI) species by forming adsorbed U-HBED surface complexes, effectively scavenging U(VI) from the solution. Considering its hard phenolate ligating groups, it is possible that HBED stabilizes U as U(IV)-HBED surface complexes, thereby inhibiting the oxidation of surface sites as discussed above. Phenolate-bearing ligands have been demonstrated to stabilize metals in redox states with a larger charge-to-radius ratio that would be unstable in uncomplexed form.^66^ Uranium concentrations in solution for the 5 μM HBED treatment were within a factor of 2 of the predicted equilibrium concentration for UO_2_. The increase in dissolved U concentrations for higher HBED concentrations is in line with the ligand-controlled dissolution of the UO_2_ phase.

#### Citrate

Uranium mobilization from UO_2_ by citrate (Figure 1D) demonstrated two distinct patterns. There is a concentration dependency up to 500 μM citrate in regards to both maximum U solution concentration as well as the mobilization rate. For the control and the 5 and 50 μM citrate treatments, the mobilized U concentrations increased marginally with the applied citrate concentration. A large increase in both maximum mobilized U concentration and mobilization rate was observed between the 50 and 500 μM treatments. Yet, at the highest ligand concentration (2 mM citrate), no further increase was observed in the maximum mobilized U concentration or the mobilization rate, suggesting a saturation of the UO_2_ surface with citrate. This is in line with a ligand-enhanced dissolution mechanism where dissolution is a function of adsorbed ligand concentration until the surface is saturated.

Both U(IV) and U(VI) complexation constants are available for citrate. Dissolved U(IV) concentrations in equilibrium with UO_2(am)_ in the presence of citrate were predicted for our experimental conditions (Figure S5). The maximum U concentrations mobilized in the various citrate treatments (Figure 1D) were plotted additionally as reference. For all citrate concentrations, the thermodynamic model underpredicted the mobilized U concentrations; for the 2 mM citrate treatment by over 2 orders of magnitude. In fact, at pH 7.0 (our experimental pH), no significant ligand-enhanced U(IV) dissolution is predicted by the model. One possible explanation for the discrepancies between experimental and modeled results could be insufficient thermodynamic data. However, another possibility previously detailed for other ligands would be that citrate is mobilizing U(VI). To assess this, aqueous U speciation in the presence of citrate was also modeled under the assumption that all mobilized U was U(VI). Under this premise, over 98% of the measured soluble U was predicted to be complexed by citrate at each ligand concentration (Figure S6). Running the same model under the assumption that all mobilized U was U(IV) (without the solubility of a UO_2_ solid phase constraining the dissolved U(IV) concentration), <1% of the soluble U was predicted to be complexed to citrate. These model predictions suggest that citrate may have mobilized U(VI) in addition to U(IV) from the UO_2_ surface.

The current findings were compared with those of Luo et al. 2011, who tested the ability of citrate to mobilize U from bioreduced sediments from the Oak Ridge, TN field site (nanoparticulate UO_2_ or U associated with Fe-oxides) in batch studies.^36^ In their work, citrate was shown to mobilize both U(IV) and U(VI).^36^ The fraction of U mobilized as U(VI) was always greater than the U(IV) fraction (∼25% U(IV) and 75% U(VI)). The applied citrate concentrations were in the same range as in this study (0.7–1.4 mM), but the total U concentrations were considerably lower: 0.8 g kg^–1^ of solids at 2 g L^–1^; ∼7 μM. While the material in Luo et al. greatly differs from the pure chemogenic mineral UO_2_ of this study, their findings of citrate mobilizing both U(IV) and, to a greater extent, U(VI) aligns with our interpretation. In both studies, models predicted U(OH)_4_ and UO_2_ citrate^–^ to be the dominant aqueous U species for U(IV) and U(VI), respectively, at the experimental pH (6.5 and 7.0 for Luo et al. and the current study, respectively), highlighting the larger influence of U(VI)–citrate complexes to those of U(IV).

The observed differences in mobilization patterns between the ligands can be attributed to numerous factors: ligand specificity for the target metal (charge-to-radius ratio, electronegativity, polarizability), geometry of the ligand, and binding moieties. U^4+^ is a hard Lewis acid, with a high ionic potential, high electronegativity, and low polarizability, implying a high affinity for hard ligating groups.^65,67^ The ligands in this study fall into two coordination categories: DFOB and HBED are both hexadentate ligands containing hydroxamate and phenolate Lewis bases, respectively, which are expected to have higher affinity for U(IV). DPA and citrate, on the other hand, are both tridentate ligands lacking hard Lewis base groups and have a predicted higher affinity for U(VI).^65^ With dissolved U concentrations for all ligands being below the calculated surface site concentration (1.3 μM U), it is feasible for surface-associated U(VI) to account for a large proportion of dissolved U concentrations, especially from softer ligands such as DPA.

Electrostatic interactions can play a role in metal-ligand complexation at mineral surfaces.^68^ The dominant speciation for each ligand at the experimental pH was modeled for correlations to mobilization rates. Each ligand exhibited a different net charge at pH 7.0, with the dominant species (DPA^2–^, H_4_DFOB^+^, H_3_HBED^–^, citrate^3–^) comprising >95% of the total speciation at pH 7.0 except for citrate (87% citrate^3–^; 13% Hcitrate^2–^). The PZC for UO_2_ is 5.4, resulting in a negative surface charge at pH 7.0, favoring the adsorption of cations.^69^ There does not appear to be a clear trend between U mobilization and net charge of the ligand at pH 7.0, suggesting that the coordination is driven more by chemical affinity than by electrostatics.

### Ligand-Induced Mobilization of Biogenic Noncrystalline U(IV)

**Figure 3:**
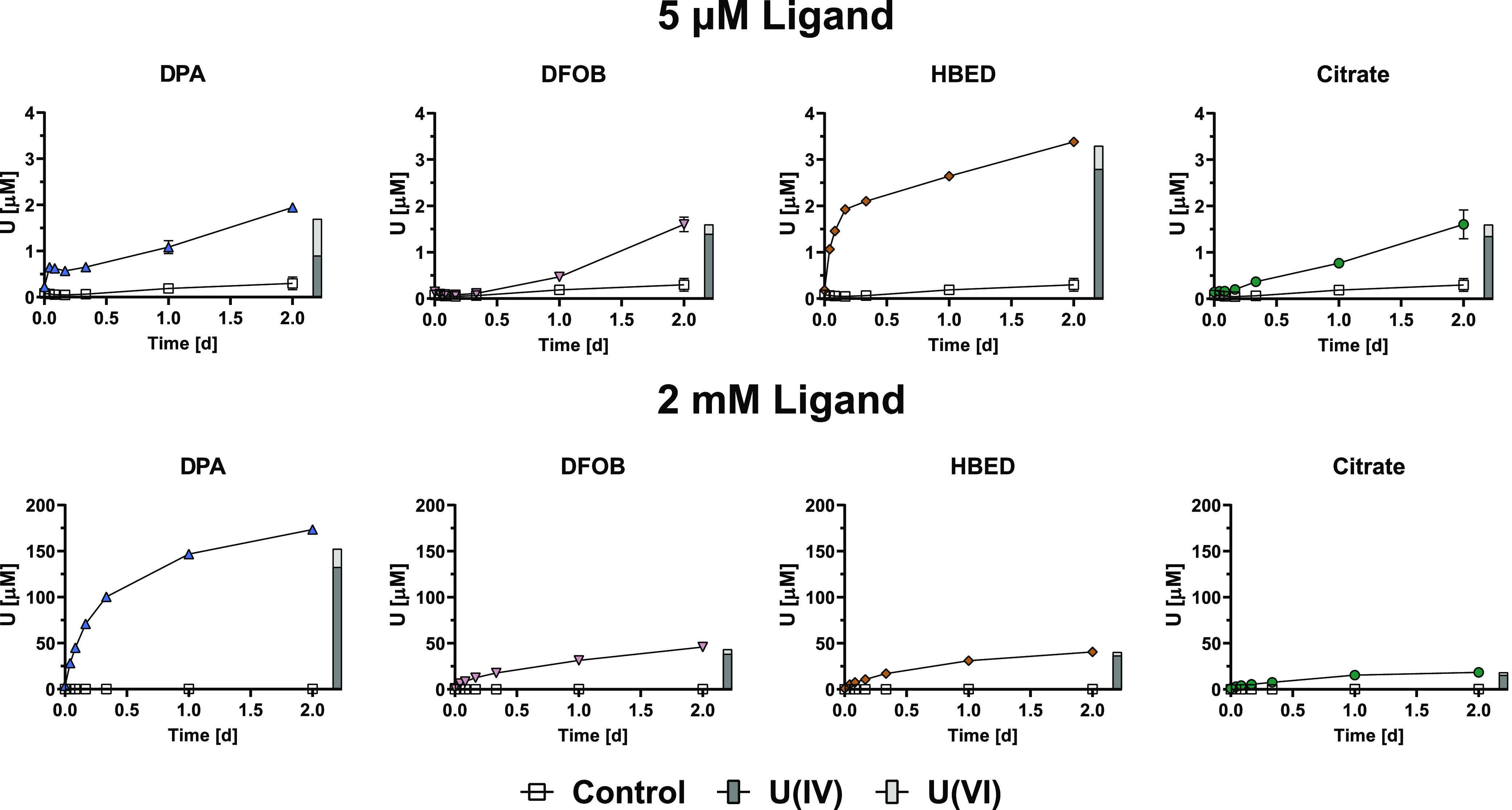
U mobilization from noncrystalline U(IV) (300 μM) induced by 5 μM and 2 mM DPA, DFOB, HBED, and citrate, at a fixed pH of 7.0. The control treatment had the same composition as other treatments except for the ligand addition. Error bars indicate standard deviations of duplicate reactors. Time *t* = 0 h corresponds to the moment of addition of an aliquot of noncrystalline U(IV) stock suspension to the solution containing pH buffer, electrolyte, and ligand. Gray bars at the 2-day timepoint correspond to ion-exchange chromatography resolution of U oxidation state in solution.

The mobilization of noncrystalline U(IV) was examined in treatments with 5 μM and 2 mM DPA, DFOB, HBED, citrate as well as in a ligand-free control treatment (Figure 3). Noncrystalline U(IV) experiments were run for a shorter duration (2 days) than UO_2_ experiments to avoid possible effects of biomass degradation on U speciation (noted by increasing dissolved U concentrations after 2 days) and because of significantly higher mobilization rates than for UO_2_. Over the 2-day experiment, the dissolved U concentration in the control treatment gradually increased, reaching up to 300 nM. The elevated control concentrations observed (notably higher than for UO_2_ by approximately 1 order of magnitude) throughout the experiment still only accounted for 0.1% of the total U in the system and were more than 1 μM lower than the final U concentrations in the 5 μM ligand treatments (nonsterile conditions are not expected to play a significant role in U mobility in these experiments). The X-ray absorption near edge structure (XANES) spectra of noncrystalline U(IV) confirmed it is predominantly U(IV) (98%) with 2% U(VI) (Figure S3). The EXAFS spectra confirmed the dominant speciation to be in fact noncrystalline U(IV) (90%) with 10% biogenic nanoparticulate UO_2_, aligning with the same speciation distribution in Alessi et al. (Figure S4).^38^

**Figure 4:**
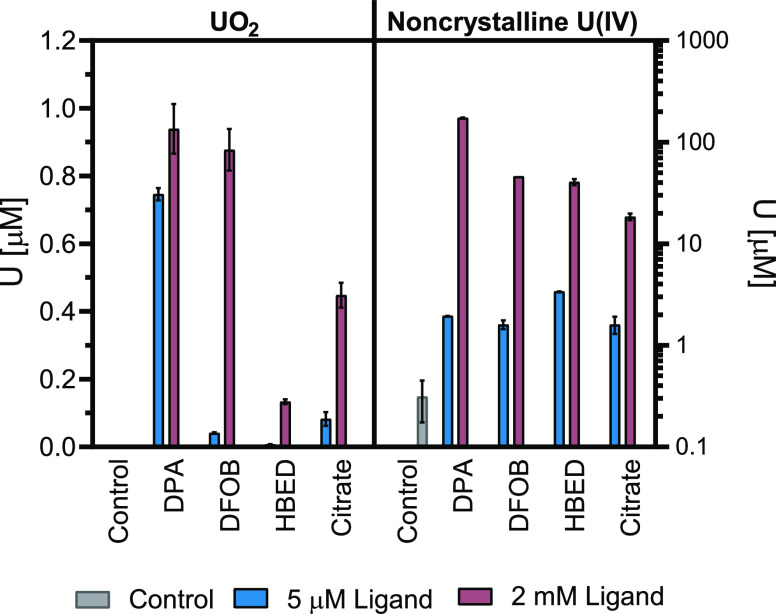
Comparison of total U concentrations mobilized from chemogenic UO_2_ and biogenic noncrystalline U(IV) in batch experiments (370 μM for UO_2_; 300 μM for noncrystalline U(IV)) by the end of each respective experiment. DPA, DFOB, HBED, and citrate are displayed at ligand concentrations of 5 μM (blue) and 2 mM (pink) in addition to control (gray) treatments in the absence of an organic ligand. UO_2_ data reflect peak U concentrations after 45 days while noncrystalline U(IV) data reflect peak U concentrations after 2 days. Total mobilized U concentrations in UO_2_ control treatments equaled 0.03 μM. Error bars indicate standard deviation of duplicate reactors.

Both the extent and rate of U mobilization were significantly higher for noncrystalline U(IV) compared to UO_2_ for all ligands. In fact, for all four ligands tested, 2 mM ligand addition with UO_2_ mobilized less U over 45 days than 5 μM ligand addition with noncrystalline U(IV) over only 2 days (Figure 4). At 5 μM ligand addition, DPA and HBED exhibited a fast initial rate of U mobilization while the rate for DFOB and citrate was slower. Yet the mobilized U concentrations from DPA, DFOB, and citrate by the end of the experiment were similar while HBED mobilized approximately twice as much U. At the higher ligand concentration of 2 mM, DPA mobilized significantly more U than the other ligands (>3.8 times more).

All four ligands showed a distinct increase in mobilized U concentration from the 5 μM to 2 mM treatment. DPA, DFOB, and citrate all exhibited significant increases in mobilized U concentration from corresponding UO_2_ experiments at matching ligand concentrations. The largest extent of mobilization was seen with DPA for both phases, while DFOB and citrate mobilized elevated levels of U which increased with ligand concentration and from UO_2_ to noncrystalline U(IV). HBED, on the other hand, was notably less potent for mobilizing U from UO_2_ (and exhibited an inhibitory effect at 5 μM HBED). However, at a 5 μM concentration it mobilized more U from noncrystalline U(IV) than any other ligand, with >1 μM U mobilized within less than 1 h. Considering the different nature of the reaction mechanism on noncrystalline U(IV) mobilization, likely a ligand exchange reaction between phosphate groups of the biomass^25^ and HBED, it is not surprising that the rate and extent of U mobilization are markedly different from UO_2_ in the presence of HBED. Additionally, if inhibitory reactions such as back-adsorption of U-HBED complexes would occur, they would be strongly influenced by the initial phase in which uranium is bound.

For the 2 mM ligand treatments, presumably, equilibrium was approached within 1–2 days (signified by stabilization of mobilized U concentrations), while for the 5 μM ligand treatments, U mobilization gradually proceeded. While all ligands mobilized substantially more at the 2 mM ligand treatment (>6% of total U for all ligands), the majority of the noncrystalline U(IV) was not mobilized with the exception of the 2 mM DPA treatment, which mobilized 57% of the total U within 2 days. The bulk of the labile noncrystalline U(IV) remaining immobilized is hypothesized to be in part due to competition between the soluble ligands and biomass functional groups for binding U. Experimental findings suggest that a larger fraction of the total U is (directly) available for complexation in noncrystalline U(IV) compared to UO_2_; a comparison based on SSA cannot be made, due to the intrinsic nature of noncrystalline U(IV) lacking a surface area. Quantitative predictions of the competition between the biomass and organic ligands for binding U would provide valuable insights. Yet, equilibrium constants for U(IV) binding to the functional groups of the biomass (vide supra) as well as to numerous organic ligands are not currently available. Furtherance of such quantitative predictions of the competition between the biomass and organic ligands for binding U could be pursued to develop effective binding constants of U for the biomass.

To gain insights regarding mobilized U speciation, an ion-exchange chromatography method was carried out at the end of each noncrystalline U(IV) experiment (2 days). Results show that while trace quantities of U(VI) are mobilized throughout each treatment, the majority of mobilized U is U(IV). The low ligand concentration (5 μM) showed a larger proportion of U(VI) than those of the higher treatment (2 mM). This aligns with the results of Xia et al. that found biogenic U(IV) with trace quantities of sorbed U(VI) to mobilize predominantly surface-adsorbed U(VI) at low ligand concentrations while at elevated ligand concentrations U(IV) comprised a larger fraction of the mobilized U.^70^ The greatest extent of aqueous U(VI) at both ligand concentrations was seen with DPA, which is expected based on the higher affinity of DPA for soft metal groups such as U(VI). Yet in the 2 mM DPA treatment the U(VI) fraction still comprised <6% of the total U in the suspension. This aligns with the XANES results which confirmed the noncrystalline U(IV) to be predominantly U(IV) with 2% U(VI) ± 5%^71^ as well as other studies which found trace quantities of U(VI) remaining after bicarbonate rinsing.^33^ These results underpin the lability of noncrystalline U(IV) to ligand-induced mobilization and the associated concerns for both U(IV) and U(VI) mobilization.

### Environmental Implications

The findings of this work highlight the stark contrast in the susceptibility, extent, and rate of ligand-induced mobilization between chemogenic UO_2_ and biogenic noncrystalline U(IV) for various organic ligands across a wide range of concentrations. The present results demonstrate the ability of organic ligands to mobilize U from a variety of reduced U solid phases under anoxic conditions.

The vulnerability of bioreduced U to reoxidation has been a known concern for decades, and subsequently, mitigation plans have aimed to account for the presence of oxidants, which could jeopardize remediation efforts. The findings of the current work highlight the concerns posed by the presence of organic ligands in the environment and the implications of ligand-induced mobilization for maintaining low U concentrations after remediation efforts, whether through direct U(IV)–ligand complexation or ligand-facilitated oxidative dissolution from residual surface-bound U(VI) present after remediation efforts. While the extent of ligand-induced U mobilization from noncrystalline U(IV) was substantially larger than from UO_2_, all ligands tested with UO_2_ still mobilized U to a level exceeding the WHO maximum contaminant level (MCL) of 30 μg L^–1^ (126 nM). Additionally, numerous countries have even stricter guidelines (i.e., Canada, 20 μg L^–1^; Germany, 10 μg L^–1^).^72,73^ Based on these findings, organic ligands (even at low concentrations) have been shown to pose a threat to even the most recalcitrant reduced U species (UO_2_) with increasing severity and concern with more labile reduced U species, such as noncrystalline U(IV).

To date, there are no equilibrium constants for the species that constitute noncrystalline U(IV), nor stability constants for U(IV) with the ligands used in this study except for citrate. The diverse group of organic ligands able to mobilize UO_2_ above drinking water standards which currently have no U(IV)-ligand stability constants exemplifies the need for further investigation into the reactivity of reduced U(IV) species with organic ligands to facilitate better preventive measures at field sites and improve modeling applications. It has been demonstrated in this study that extrapolating a ligand’s ability to mobilize U from U(IV) species based on trends in complexation strength for other metals should be avoided, as was exhibited by the strong Fe(III)-chelating ligand HBED, which did not pose the largest threat for U(IV)-containing solids. Other organic ligands must be considered for their role in enhancing U solubility, such as illustrated in this study with DPA.

## Supporting Information Available

Description of UO_2_ synthesis and characterization, noncrystalline U(IV) XANES and EXAFS analysis, and ion-exchange chromatography method, Widdel Low Phosphate medium composition, thermodynamic stability constants, overview of U mobilization rates from UO_2_, XRD pattern of UO_2_, SEM image of UO_2_, XANES and EXAFS of noncrystalline U(IV), model predictions for U(VI) in UO_2_ experiments, UO_2_ mobilization rates from DFOB plotted against adsorbed ligand concentration, zoom-in version of Figure 1 for seeing HBED concentration effects, and modeled equilibrium speciation of UO_2_ in the presence of citrate alongside experimental values (PDF).
