# Supplementary material for: Ligand-Induced U Mobilization from Chemogenic Uraninite and Biogenic Noncrystalline U(IV) under Anoxic Conditions
Source: Environ Sci Technol. 2022 May 6;56(10):6369–79. doi: 10.1021/acs.est.1c07919 (PMC9118557; doi:10.1021/acs.est.1c07919)
Supplement: Supplementary file 1 — es1c07919_si_001.pdf [file es1c07919_si_001.pdf]

Ligand-Induced U Mobilization from Chemogenic Uraninite and Biogenic Noncrystalline U(IV) under Anoxic Conditions

Kyle J. Chardi[a], Anshuman Satpathy[b], Walter D. C. Schenkeveld[c*], Naresh Kumar[c], Vincent Noël[d], Stephan M. Kraemer[a], Daniel E. Giammar[e]

[a] Centre for Microbiology and Environmental Systems Science, Department for Environmental Geosciences, University of Vienna, Josef-Holaubek-Platz 2 1090 Vienna, Austria
[b] Department of Civil and Environmental Engineering and Earth Sciences, University of Notre Dame, Note Dame, Indiana 46556, United States
[c] Soil Chemistry and Chemical Soil Quality Group, Wageningen University and Research, Droevendaalsesteeg 3, 6708 PB Wageningen, the Netherlands
[d] Stanford Synchrotron Radiation Lightsource, SLAC National Accelerator Laboratory, 2575 Sand Hill Road, 94025 Menlo Park, California, United States
[e] Department of Energy, Environmental, and Chemical Engineering, One Brookings Drive, Washington University, St. Louis, Missouri 63130, United States

* Corresponding author: walter.schenkeveld@wur.nl

Supporting Information

14 pages
4 text sections
3 tables
8 figures

This supporting information contains the following:

Text S1. UO_2 synthesis

Text S2. UO_2 characterization

Text S3. X-ray absorption spectroscopy of noncrystalline U(IV)

Text S4. Ion-exchange chromatography method

Table S1. Basal medium composition of Widdel Low Phosphate (WLP) used for synthesis of noncrystalline U.

Table S2. Thermodynamic stability constants (T = 25° C, I = 0) for aqueous and solid phase reactions as used in Visual MINTEQ.

Table S3. Mobilization rates for DFOB, DPA, HBED, and citrate of UO_2 at treatment concentrations of 5 μM, 50 μM, 500 μM, and 2 mM with a fixed pH of 7.0 for 330 μM U (0.1 g L^{-1} UO_2) with SSA of 4.9 m^2 g^{-1}. All rates calculated in two stages: from 0 – 2 d and from 2 – 23 d, corresponding to Figure 2A and Figure 2B, respectively.

Figure S1. XRD pattern for UO_2. Red line indicates ICDD reference pattern for UO_2 (ICDD 00-041-1422). The broad peak from 8º to 17º 2θ is attributed to the anoxic dome over the sample as it was analyzed.

Figure S2. SEM image of UO_2.

Figure S3. U *LIII*-edge XANES of synthesized noncrystalline U(IV) (black lines), and LC-LS fit results (red lines). Sample underwent 50 mM bicarbonate rinsing prior to analysis to align with conditions at the start of ligand mobilization experiments. The values of these best LC-LS fits, normalized at 100%, are reported on the right with *R_f (R factor) calculated as $R_f = \Sigma\, [k^3 X(k)_{exp} - k^3 X(k)_{calc}]^2 / \Sigma\, [k^3 X(k)_{exp}]^2$. Experimental XANES spectra of the model compounds of noncrystalline U(IV)- and U(VI)-sorbed, used for this LC-LS fitting analysis are displayed above and below the sample data, respectively.

Figure S4. U LIII-edge EXAFS of synthesized noncrystalline U(IV) (black lines), and LC-LS fit results (red lines). Sample underwent 50 mM bicarbonate rinsing prior to analysis to align with conditions at the start of ligand mobilization experiments. The values of these best LC-LS fits, normalized at 100%, are reported on the right with *R_f (R factor) calculated as $Rf = \Sigma\, [k^3 X (k)_{exp} - k^3 X (k)_{calc}]^2 / \Sigma\, [k^3 X (k)_{exp}]^2$. Experimental EXAFS spectra of the model compounds of nano-UO_2 and noncrystalline U(IV) used for this LC-LS fitting analysis are displayed above and below the sample, respectively.

Figure S5. Predicted aqueous U(IV) speciation under experimental conditions (6.6 mM NaCl and 10 mM MOPS) in equilibrium with UO_2 and 2 mM citrate based off of the constants provided in Table S2. Data points reflect the highest extent of mobilization seen in corresponding citrate experiments (Figure 1D). Model prepared in Visual MINTEQ.

Figure S6. Proportion of experimentally found total mobilized U from ligand batch experiments (Figure 1) with UO_2 which thermodynamically are predicted to be complexed to each

respective ligand (with the exception of HBED which does not have reported U(VI)-ligand stability constants) with U input as U(VI). Stability constants are summarized in (Table S2). Model prepared in Visual MINTEQ.

Figure S7. UO_2 mobilization rates from DFOB plotted as a function of adsorbed ligand concentration for the mobilization rates from 0 – 2 d and 2 – 23 d (as shown in Figure 2) by utilizing the isotherm parameters from Frazier et al. for each of the tested ligand concentrations. The slope of the linear regression line or k_L was reported for both stages in addition to the coefficient of determination or R^2.

Figure S8 HBED-induced mobilization of UO_2 results. These results mimic those shown in Figure 1C but scaled to the extent of HBED mobilization to see in better resolution inhibitory effects seen at 5 µM ligand concentration.

Text S1. UO_2 synthesis

UO_2 was synthesized following the protocol described in Ulrich et al., 2008 and summarized below. Uranyl nitrate was heated at 275 °C under oxic conditions for 72 hours in a muffle furnace to remove nitrates and produce $UO_{3(s)}$ (schoepite).

The freshly synthesized $UO_{3(s)}$ was dissolved in 0.5 M HCl to yield a UO_2Cl_2 aqueous solution. This solution was mixed with 30 mL of H_2O_2 in a 3 L HDPE reactor filled to volume with ultra-pure water (UPW) and continuously stirred for 72 hours. A pale-yellow precipitate, uranium peroxide, was then allowed to settle at the bottom of the reactor and the supernatant was discarded. The resultant slurry was dialyzed against UPW for an additional 72 hours. Every 24 hours electrical conductivity (EC) was measured (as an indicator for high ionic concentrations) in the UPW and replaced with fresh UPW. After 72 hours the EC was found to be nearly zero and dialysis was subsequently stopped.

The uranium peroxide precipitate was transferred to a reactor tube and frozen to -80 °C prior to being freeze dried for 96 hours to evaporate residual water without causing any structural changes to the uranium peroxide.

Dried uranium peroxide was then reduced to UO_2 in a stainless-steel reactor in the presence of $H_{2(g)}$ at 400 °C for 4 hours by use of a cartridge heater and temperature

controller. To ensure a continuous supply of $H_{2(g)}$, the reactor was connected on the influent-end to a pressurized hydrogen cylinder and a tubing from the effluent end was inserted in a beaker of UPW to gauge the continuous flow based off of the presence of bubbles. After 4 hours of reaction, the heater was turned off and allowed to cool while $H_{2(g)}$ continued to flow to maintain anoxic conditions within the reactor. Once reaching room temperature, the reactor was transferred to an anaerobic chamber (Coy Laboratory Products Inc.) containing a gas mixture of 95% $N_{2(g)}$ and 5% $H_{2(g)}$. The UO_2 was stored in an amber glass bottle until use.

Text S2. UO_2 characterization

X-ray powder diffraction (XRD, Bruker d8 Advance powder diffractometer with a Cu Kα X-ray source and a LYNXEYE XE energy-dispersive strip detector) was used to characterize the freshly prepared UO_2. To prevent oxidation of the material during the measurement, the sample was loaded onto a silicon zero diffraction plate and enclosed in an anoxic dome inside an oxygen-free glove box. The dome remained over the sample throughout analysis. The resultant broad peak from 8º to 17º 2θ in the pattern is attributed to the dome. The XRD pattern for UO_2 aligned well with the International Center for Diffraction Data ICDD reference pattern (ICDD 00-041-1422) (Figure S1).

Text S3. X-ray absorption spectroscopy of noncrystalline U(IV)

X-ray absorption spectroscopy was carried out on the synthesized noncrystalline U(IV) following the same 50 mM bicarbonate rinsing and subsequent anoxic water rinsing steps prior to the start of ligand remobilization experiments. Uranium *LIII*-edge X-ray absorption spectrum of the synthesized noncrystalline U(IV) was collected at beamline

4-1 at the Stanford Synchrotron Radiation Lightsource (SSRL). The energy of the X-ray beam was selected using a Si(220)φ=0 double-crystal monochromator detuned by 30% at 17,700 eV to minimize harmonics. Synthesized noncrystalline U(IV) sample was mounted on a cryostat sample rod inside an N_2-purged glove bag and brought to the beamline in a liquid nitrogen bath before being rapidly transferred into the liquid N cryostat. Calibration was monitored during each sample scan by collecting the Y *K*-edge (17,038.4 eV) in double transmission setup. The fluorescence signal of the synthesized noncrystalline U(IV) sample was monitored using a 30-element germanium detector. Soller slits were used to increase the signal-to-noise ratio. 9 scans were recorded, and beam damage was not observed during measurement of sequential scans, as expected.[1] All spectra were averaged and normalized using the ATHENA software.[2] Radial distribution functions around the U absorber were obtained by Fast-Fourier-transformation of the k^3-weighted experimental $\chi(k)$ function using a Kaiser-Bessel apodization window with the Bessel weight fixed at 2.5.

The U oxidation state in the synthesized noncrystalline U(IV) was verified using U *LIII*-edge X-ray Absorption Near Edge Structure (XANES) data (Figure S3). U *LIII*-edge XANES spectrum was analyzed by linear combination-least squares (LC-LS) fitting using the ATHENA software.[2] Linear coefficients were only constrained to be positive. We consider the accuracy of this fitting procedure to about ±5% of the stated values for each individual contribution according to the error calculated by Singer et al. 2009.[3] Components contributing less than 5% are thus considered not significant. To enable comparison of spectra measured at different beam lines, the vertical limiting aperture was set sufficiently small to ensure that the spectrometer resolution was much lower than the energy corresponding to the core-hole lifetime (c. 9 eV). The U model compounds used for the XANES LC-LS fitting analysis include noncrystalline U(VI)-

sorbed and U(IV)-sorbed (Figure S3).[4] The exact similarity of the energy position of the U *LIII*-edge XANES white-line of the synthesized noncrystalline U(IV)with that of U(IV) model compound, as well the results of LS-LC fits, confirm the near-purity of U(IV) (with U(VI) <5%, which is below the detection limit; Figure S3).[3]

Then, U *LIII*-edge X-ray Extended Absorption Fine Structure (EXAFS) spectrum of the synthesized noncrystalline U(IV) were analyzed by LC-LS fitting procedure to quantify the proportion of noncrystalline and crystalline U(IV). The U model compounds used for the LC-LS fitting analysis include noncrystalline U(IV) (Bone et al. 2017) and nano-UO_2 (Massey et al. 2014; Figure S4).[5, 6] Our result confirms that U *LIII*-edge EXAFS spectrum of the synthesized noncrystalline U(IV) compound is mainly composed of noncrystalline U(IV) (90%), and 10% biogenic nanoparticulate UO_2. This level of nanoparticulate UO_2 present in the synthesis of noncrystalline U(IV) has previously been documented in Alessi et al. 2012.[7]

Text S4. Ion-exchange chromatography method

Dowex 1 x 8 (200 – 400 mesh) chloride form anion exchange resin was packed under anaerobic conditions in polypropylene chromatography columns (Poly-Prep, Bio-Rad). The U oxidation state was resolved by first stabilizing pre-filtered (0.2 μm cellulose acetate) anoxic batch suspension samples to 4.5 M HCl. Samples were then loaded into resin-packed columns which were pretreated with 10 pore volumes of 0.1 M HCl followed by 10 pore volumes of 4.5 M HCl. The U(VI) fraction is selectively eluted by addition of 10 pore volumes of 0.1 M HCl and then the U(IV) fraction eluted by addition

of 10 pore volumes of 4.5 M HCl. Samples were measured on ICP-MS after dilution in 1% HNO_3.

Table S1. Basal medium composition of Widdel Low Phosphate (WLP) used for synthesis of noncrystalline U(IV).

Compound	WLP (mM)
$CaCl_2 \cdot 2H_20$	0.68
KCl	6.71
KH_2PO_4	0.22
$MgCl_2 \cdot 6H_20$	2.46
NaCl	85.56
NH_4Cl	4.67
$NaHCO_3$	30
PIPES	20
pH	7.3

Table S2. Thermodynamic stability constants (T = 25° C, I = 0) for aqueous and solid phase reactions as used in Visual MINTEQ.

Reaction	Log K	Source
**U Hydrolysis and Redox**		
$U^{4+} + H_2O \rightleftharpoons UOH^{3+} + H^+$	-0.4	1
$U^{4+} + 2H_2O \rightleftharpoons U(OH)_2^{2+} + 2H^+$	-1.1	1
$U^{4+} + 3H_2O \rightleftharpoons U(OH)_3^{+} + 3H^+$	-4.7	1
$U^{4+} + 4H_2O \rightleftharpoons U(OH)_{4(aq)} + 4H^+$	-10	1
$U^{4+} + 2H_2O \rightleftharpoons UO_{2(am,\ hyd)} + 4H^+$	-1.5	1
$UO_2^{2+} + H_2O = UO_2OH^+ + H^+$	-5.25	1
$UO_2^{2+} + 2H_2O = UO_2(OH)_{2(aq)} + 2H^+$	-12.15	1
$UO_2^{2+} + 3H_2O = UO_2(OH)_3^- + 3H^+$	-20.25	1
$UO_2^{2+} + 4H_2O = UO_2(OH)_4^{2-} + 4H^+$	-32.4	1
$2UO_2^{2+} + H_2O = (UO_2)_2OH^{3+} + H^+$	-2.7	1
$2UO_2^{2+} + 2H_2O = (UO_2)_2(OH)_2^{2+} + 2H^+$	-5.62	1
$3UO_2^{2+} + 4H_2O = (UO_2)_3(OH)_4^{2+} + 4H^+$	-11.9	1
$3UO_2^{2+} + 5H_2O = (UO_2)_3(OH)_5^{+} + 5H^+$	-15.55	1
$3UO_2^{2+} + 7H_2O = (UO_2)_3(OH)_7^{-} + 7H^+$	-32.2	1
$4UO_2^{2+} + 7H_2O = (UO_2)_4(OH)_7^{+} + 7H^+$	-21.9	1
**U Inorganic Anion Complexation**		
$U^{4+} + Cl^- \rightleftharpoons UCl^{3+}$	1.72	1
$U^{4+} + 2Cl^- \rightleftharpoons UCl_2^{2+}$	0.06	1

**Citrate Hydrolysis**		
$Citrate^{3-} + H^{+} \rightleftharpoons HCitrate^{2-}$	6.4	2
$Citrate^{3-} + 2H^{+} \rightleftharpoons H_2Citrate^{-}$	11.2	2
$Citrate^{3-} + 3H^{+} \rightleftharpoons H_3Citrate$	14.3	2
**U(IV)-Citrate Complexation**		
$Citrate^{3-} + U^{4+} \rightleftharpoons UCitrate^{+}$	12.8	3
$2Citrate^{3-} + U^{4+} \rightleftharpoons U(Citrate)_2^{2+}$	19.5	2
**U(VI)-Citrate Complexation**		
$Citrate^{3-} + UO_2^{2+} \rightleftharpoons UO_2Citrate^{-}$	8.96	2
$2Citrate^{3-} + 2UO_2^{2+} \rightleftharpoons (UO_2)_2(Citrate)^{2-}$	21.30	2
$HCitrate^{2-} + UO_2^{2+} \rightleftharpoons UO_2(Hcitrate)_{(aq)}$	5.00	2
**DPA Hydrolysis**		
$DPA^{2-} + H^{+} \rightleftharpoons HDPA^{-}$	4.53	4
$DPA^{2-} + 2H^{+} \rightleftharpoons H_2DPA_{(aq)}$	6.60	4
$DPA^{2-} + 3H^{+} \rightleftharpoons H_3DPA^{+}$	8.62	4
**U(VI)-DPA Complexation**		
$DPA^{2-} + UO_2^{2+} \rightleftharpoons UO_2DPA_{(aq)}$	9.82	5
$2DPA^{2-} + UO_2^{2+} \rightleftharpoons UO_2DPA_2^{2-}$	15.86	5
**DFOB Hydrolysis**		
$DFOB^{3-} + H^{+} \rightleftharpoons HDFOB^{2-}$	11.48	6
$DFOB^{3-} + 2H^{+} \rightleftharpoons H_2DFOB^{-}$	21.46	6
$DFOB^{3-} + 3H^{+} \rightleftharpoons H_3DFOB_{(aq)}$	30.66	6
$DFOB^{3-} + 4H^{+} \rightleftharpoons H_3DFOB^{+}$	38.96	6
**U(VI)-DFOB Complexation**		
$HDFOB^{2-} + UO_2^{2+} \rightleftharpoons UO_2(HDFOB)_{(aq)}$	23.78	7

(1) Guillaumont et al. 2003[8]
(2) Hummel et al. 2007[9]
(3) Bonin et al. 2008[10]
(4) Bombi et al. 2009[11]
(5) Xu et al.2013[12]
(6) Smith et al. 2004[13]
(7) Mullen et al. 2007[14]

Table S3. Mobilization rates for DFOB, DPA, HBED, and citrate of UO_2 at treatment concentrations of 5 μM, 50 μM, 500 μM, and 2 mM with a fixed pH of 7.0 for 330 μM U (0.1 g L^{-1} UO_2) with SSA of 4.9 m^2 g^{-1}. All rates calculated in two stages: from 0 – 2 d and from 2 – 23 d, corresponding to Figure 2A and Figure 2B, respectively.

0 - 2 d

Treatment	nM h^{-1}	nmol h^{-1} g^{-1}	nmol h^{-1} m^{-2}	R^2
Control	0.0	0.1	0.03	0.11
DPA - 5 μM	2.0	20.4	4.2	0.63
DPA - 50 μM	3.7	36.7	7.5	0.95
DPA - 500 μM	2.5	24.7	5.0	0.82
DPA - 2 mM	2.4	24.5	5.0	0.63
DFOB - 5 μM	0.0	0.0	0.0	0.05
DFOB - 50 μM	0.8	8.3	1.7	0.72
DFOB - 500 μM	1.5	14.8	3.0	0.92
DFOB - 2 mM	1.7	17.2	3.5	0.98
HBED - 5 μM	0.0	0.0	0.0	0.63
HBED - 50 μM	0.2	2.3	0.5	0.96
HBED - 500 μM	0.4	3.9	0.8	0.85
HBED - 2 mM	0.4	3.8	0.8	0.91
Citrate - 5 μM	0.1	0.8	0.2	0.36
Citrate - 50 μM	0.1	1.0	0.2	0.14
Citrate - 500 μM	0.9	8.8	1.8	0.61
Citrate - 2 mM	1.2	12.4	2.5	0.75

2 - 23 d

Treatment	nM h^{-1}	nmol h^{-1} g^{-1}	nmol h^{-1} m^{-2}	R^2
Control	0.0	0.1	0.03	0.70
DPA - 5 μM	0.0	0.0	0.0	0.99
DPA - 50 μM	0.5	4.6	0.9	1.00
DPA - 500 μM	0.7	6.8	1.4	0.98
DPA - 2 mM	0.7	7.5	1.5	0.96
DFOB - 5 μM	0.1	0.6	0.1	0.96
DFOB - 50 μM	0.4	3.6	0.7	0.95
DFOB - 500 μM	0.8	8.3	1.7	0.98
DFOB - 2 mM	1.1	10.9	2.2	1.00

HBED - 5 μM	0.0	0.1	0.0	0.70
HBED - 50 μM	0.1	0.9	0.2	0.97
HBED - 500 μM	0.2	1.6	0.3	0.98
HBED - 2 mM	0.2	1.7	0.4	0.99
Citrate - 5 μM	0.1	0.6	0.1	1.00
Citrate - 50 μM	0.1	1.2	0.2	0.97
Citrate - 500 μM	0.5	4.9	1.0	0.99
Citrate - 2 mM	0.5	4.7	1.0	0.97

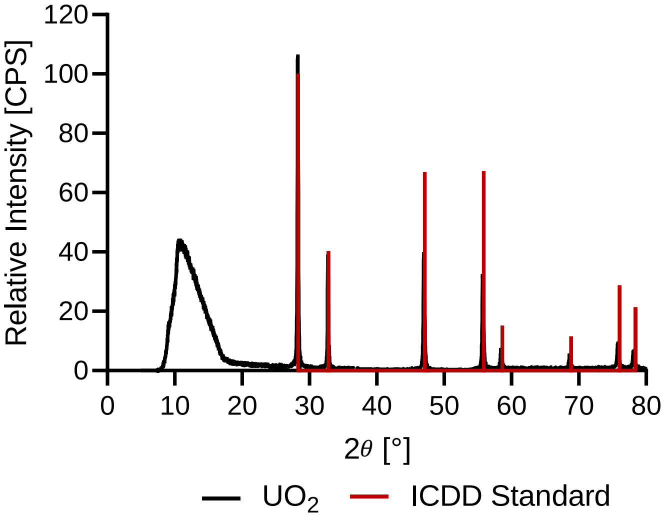

Figure S1. XRD pattern for UO_2. Red line indicates ICDD reference pattern for UO_2 (ICDD 00-041-1422). The broad peak from 8º to 17º 2θ is attributed to the anoxic dome over the sample as it was analyzed.

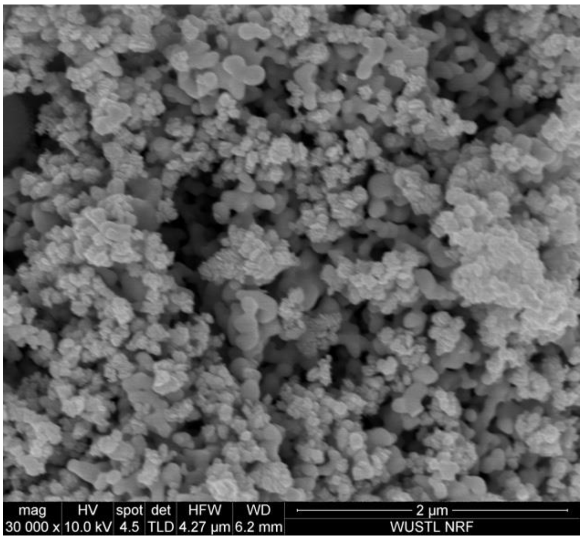

Figure S2. SEM image of UO_2.

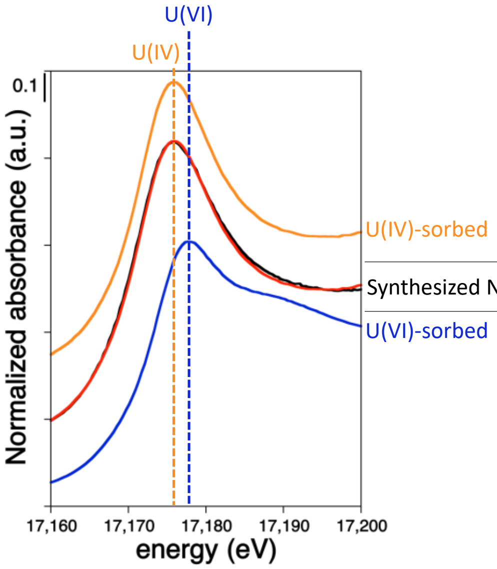

LC-LS fits results

	U(IV)	U(VI)	Sum	Rf*(10^{-4})
Synthesized Non-crystalline U(IV)	98%	2%	100%	8.7

Figure S3. U *LIII*-edge XANES of synthesized noncrystalline U(IV) (black lines), and LC-LS fit results (red lines). Sample underwent 50 mM bicarbonate rinsing prior to analysis to align with conditions at the start of ligand mobilization experiments. The values of these best LC-LS fits, normalized at 100%, are reported on the right with *R_f (R factor) calculated as $R_f = \Sigma [k^3 X(k)_{exp} - k^3 X(k)_{calc}]^2 / \Sigma [k^3 X(k)_{exp}]^2$. Experimental XANES spectra of the model compounds of noncrystalline U(IV)- and U(VI)-sorbed, used for this LC-LS fitting analysis are displayed above and below the sample data, respectively.

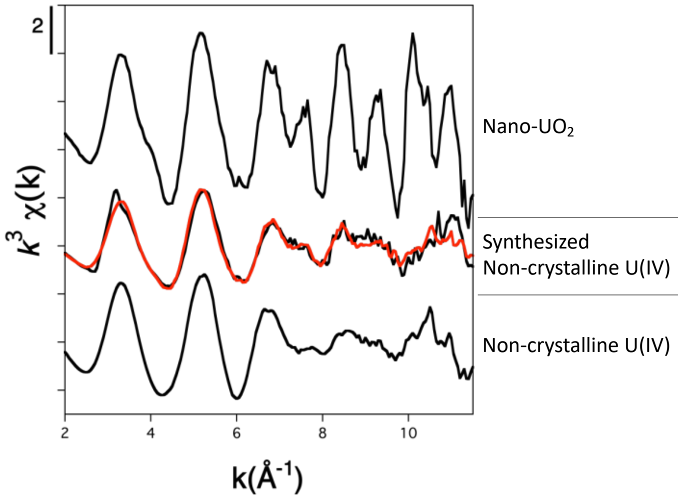

LC-LS fits results

	Non-crystalline U(IV)	Nano-UO$_2$	Sum	Rf*(10^{-2})
Synthesized Non-crystalline U(IV)	90%	10%	100%	7.1

Figure S4. U *LIII*-edge EXAFS of synthesized noncrystalline U(IV) (black lines), and LC-LS fit results (red lines). Sample underwent 50 mM bicarbonate rinsing prior to analysis to align with conditions at the start of ligand mobilization experiments. The values of these best LC-LS fits, normalized at 100%, are reported on the right with *R_f (R factor) calculated as $Rf = \Sigma [k^3 X(k)_{exp} - k^3 X(k)_{calc}]^2 / \Sigma [k^3 X(k)_{exp}]^2$. Experimental EXAFS spectra of the model compounds of nano-UO_2 and noncrystalline U(IV) used for this LC-LS fitting analysis are displayed above and below the sample, respectively.

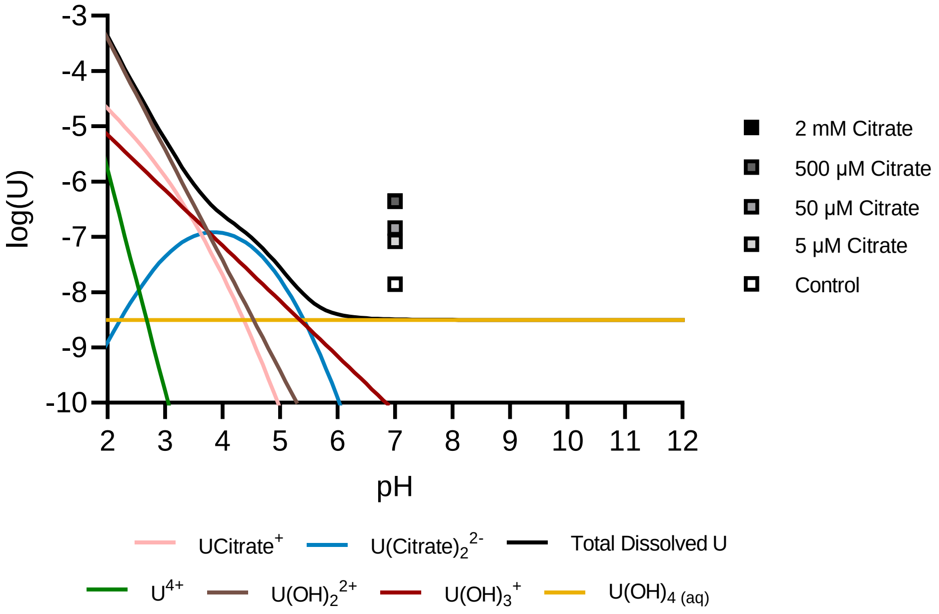

Figure S5. Predicted aqueous U(IV) speciation under experimental conditions (6.6 mM NaCl and 10 mM MOPS) in equilibrium with UO_2 and 2 mM citrate based off of the constants provided in Table S2. Data points reflect the highest extent of mobilization seen in corresponding citrate experiments (Figure 1D). Model prepared in Visual MINTEQ.

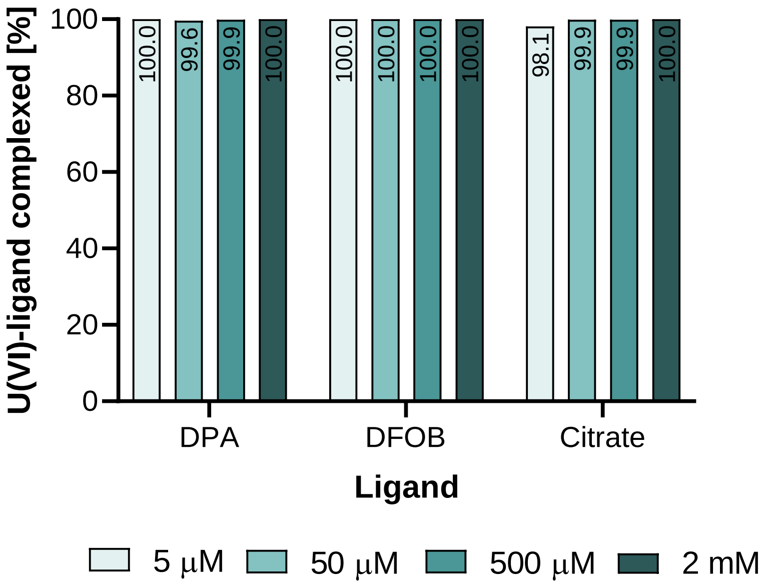

Figure S6. Proportion of experimentally found total mobilized U from ligand batch experiments (Figure 1) with UO_2 which thermodynamically are predicted to be complexed to each respective ligand (with the exception of HBED which does not have reported U(VI)-ligand stability constants) with U input as U(VI). Stability constants are summarized in (Table S2). Model prepared in Visual MINTEQ.

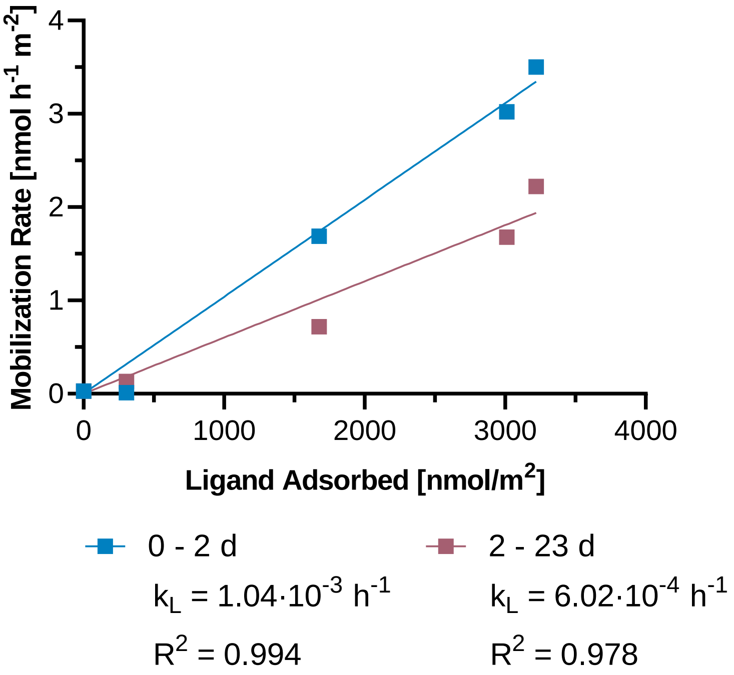

Figure S7. UO_2 mobilization rates from DFOB plotted as a function of adsorbed ligand concentration for the mobilization rates from 0 – 2 d and 2 – 23 d (as shown in Figure 2) by utilizing the isotherm parameters from Frazier et al. for each of the tested ligand concentrations. The slope of the linear regression line or k_L was reported for both stages in addition to the coefficient of determination or R^2.

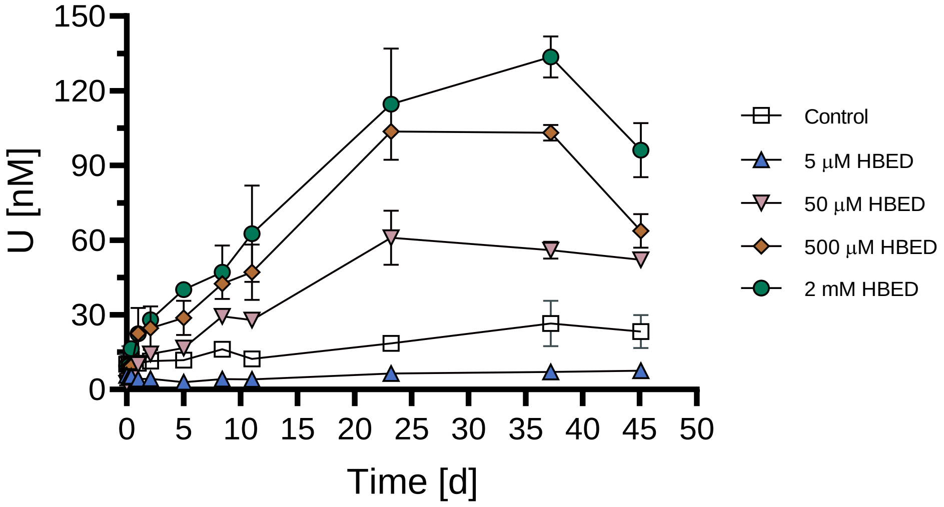

Figure S8. HBED-induced mobilization of UO_2 results. These results mimic those shown in Figure 1C but scaled to the extent of HBED mobilization to see in better resolution inhibitory effects seen at 5 µM ligand concentration.

References

1. Alessi, D. S.; Uster, B.; Borca, C. N.; Grolimund, D.; Bernier-Latmani, R., Beam-induced oxidation of monomeric U (IV) species. *Journal of Synchrotron Radiation* **2013,** *20*, (1), 197-199.
2. Ravel, B.; Newville, M., ATHENA, ARTEMIS, HEPHAESTUS: data analysis for X-ray absorption spectroscopy using IFEFFIT. *Journal of synchrotron radiation* **2005,** *12*, (4), 537-541.
3. Singer, D. M.; Farges, F.; Brown Jr, G. E., Biogenic nanoparticulate UO2: Synthesis, characterization, and factors affecting surface reactivity. *Geochimica et Cosmochimica Acta* **2009,** *73*, (12), 3593-3611.
4. Noël, V.; Boye, K.; Kukkadapu, R. K.; Li, Q.; Bargar, J. R., Uranium storage mechanisms in wet-dry redox cycled sediments. *Water research* **2019,** *152*, 251-263.
5. Bone, S. E.; Dynes, J. J.; Cliff, J.; Bargar, J. R., Uranium (IV) adsorption by natural organic matter in anoxic sediments. *Proceedings of the National Academy of Sciences* **2017,** *114*, (4), 711-716.
6. Massey, M. S.; Lezama-Pacheco, J. S.; Jones, M. E.; Ilton, E. S.; Cerrato, J. M.; Bargar, J. R.; Fendorf, S., Competing retention pathways of uranium upon reaction with Fe (II). *Geochimica et cosmochimica acta* **2014,** *142*, 166-185.
7. Alessi, D. S.; Uster, B.; Veeramani, H.; Suvorova, E. I.; Lezama-Pacheco, J. S.; Stubbs, J. E.; Bargar, J. R.; Bernier-Latmani, R., Quantitative separation of monomeric U (IV) from UO2 in products of U (VI) reduction. *Environmental science & technology* **2012,** *46*, (11), 6150-6157.
8. Guillaumont, R.; Mompean, F. J., *Update on the chemical thermodynamics of uranium, neptunium, plutonium, americium and technetium*. Elsevier Amsterdam: 2003; Vol. 5.
9. Hummel, W.; Mompean, F. J.; Illemassène, M.; Perrone, J., *Chemical thermodynamics of compounds and complexes of U, Np, Pu, Am, Tc, Se, Ni and Zr with selected organic ligands*. Elsevier Amsterdam: 2005; Vol. 9.
10. Bonin, L.; Cote, G.; Moisy, P., Speciation of An (IV)(Pu, Np, U and Th) in citrate media. *Radiochimica Acta* **2008,** *96*, (3), 145-152.
11. Bombi, G. G.; Aikebaier, R.; Dean, A.; Di Marco, V. B.; Marton, D.; Tapparo, A., Complexation of 2, 6-pyridinedicarboxylic and 2, 6-pyridinediacetic acids towards aluminium (III) and iron (III). *Polyhedron* **2009,** *28*, (2), 327-335.
12. Xu, C.; Tian, G.; Teat, S. J.; Rao, L., Complexation of U (VI) with dipicolinic acid: thermodynamics and coordination modes. *Inorganic chemistry* **2013,** *52*, (5), 2750-2756.
13. Smith, R.; Martell, A.; Motekaitis, R., NIST standard reference database 46. *NIST Critically Selected Stability Constants of Metal Complexes Database Ver* **2004,** *2*.
14. Mullen, L.; Gong, C.; Czerwinski, K., Complexation of uranium (VI) with the siderophore desferrioxamine B. *Journal of Radioanalytical and Nuclear Chemistry* **2007,** *273*, (3), 683-688.
